# Determinants of Exposure to Secondhand Smoke Among Vietnamese Adults: California Vietnamese Adult Tobacco Use Survey, 2007–2008

**DOI:** 10.5888/pcd11.130327

**Published:** 2014-05-15

**Authors:** Whitney L. Webber, Brianna van Erp, Pamela Stoddard, Janice Y. Tsoh

**Affiliations:** Author Affiliations: Brianna van Erp, Pamela Stoddard, Santa Clara County Public Health Department, San Jose, California; Janice Y. Tsoh, University of California Department of Psychiatry, San Francisco, California, and Asian American Research Center, San Francisco, California.

## Abstract

Because smoking rates are high among Vietnamese men, we used data from the 2007–2008 California Vietnamese Adult Tobacco Use Survey to estimate secondhand smoke exposure and associated risk factors among Vietnamese nonsmokers. Thirty percent of nonsmokers were exposed to secondhand smoke (SHS) at home, 8% at work, 52% in bars, and 67% on a college campus. At home, odds of SHS exposure were greater for women than for men and for adults aged less than 40 years than for older adults. Odds of SHS exposure were higher for former smokers at work (among employed men) and among men when in bars. Future interventions should consider sex, age, and smoking history in efforts to prevent SHS exposure among Vietnamese adults.

## Objective

Secondhand smoke (SHS) exposure is associated with illness and death (1). Given high smoking rates among Vietnamese men (2–5), SHS exposure may be high in the Vietnamese population; however, research on this population is limited. As communities seek to reduce SHS exposure among vulnerable populations through programs such as the Communities Putting Prevention to Work (CPPW) initiative of the Centers for Disease Control and Prevention (CDC) (6), understanding SHS exposure among Vietnamese adults is timely and necessary for targeting interventions. This study aimed to estimate SHS exposure and associated risk factors among Vietnamese adults.

## Methods

Data were from the 2007–2008 California Vietnamese Adult Tobacco Use Survey (CVATUS), a landline telephone survey representative of Vietnamese adults in California conducted in Vietnamese and English with a 63.5% participation rate. Details of the survey, which was approved by the institutional review board at the University of California, San Francisco, have been described previously (7).

SHS exposure at home was coded “yes” if respondents indicated that anyone living in the household smokes cigarettes now, or smoking was allowed inside the home, or anyone ever smoked inside the home. Employed respondents who primarily worked outdoors or worked inside a building where someone had smoked in the past 2 weeks were classified as exposed to SHS at work. Because of very low workplace SHS exposure among women (2%), only employed men were included in the workplace analysis. Respondents who visited a bar, tavern, nightclub, or restaurant in the past 12 months with smoking inside were classified as exposed to SHS in bars. Respondents enrolled in a course on a college campus who reported being exposed to other people’s tobacco smoke on campus indoors or outdoors in the past 2 weeks were classified as exposed to SHS at college.

Independent variables included sex, age, education, English proficiency, years in the United States, children in the home, smoking history, SHS knowledge, and SHS attitudes. Respondents were coded as knowledgeable of SHS health effects if they responded correctly to each of 3 questions relating to the health effects of SHS exposure.

The analytic sample included 1,887 nonsmokers (former and never smokers) who provided complete data. Current smokers were excluded. We present descriptive statistics (weighted) and used weighted logistic regression to estimate the odds of SHS exposure in the above settings.

## Results

Almost half of nonsmoking adults were aged 40 or older (48%) (Table 1). Nearly 4 in 5 adults (79%) had limited English proficiency. Most adults (80%) were knowledgeable about the health effects of SHS exposure, and 90% had a favorable attitude toward California’s law prohibiting smoking in bars. A substantial percentage had SHS exposure at home (30%), work (8%), bars (52%), and college (67%). Most adults did not allow smoking inside their home (95%) (not shown). Among these, only 6% reported anyone had ever smoked inside their home (not shown).

**Table 1:** Characteristics of Vietnamese Adult Nonsmokers, California Vietnamese Adult Tobacco Use Survey, 2007–2008 (unweighted n = 1,887)

Characteristic	% (unweighted n)^a^
**Sex**	
Female	58 (1,066)
Male	43 (821)
**Age, y**	
<40	53 (424)
≥40	48 (1,447)
**Education**	
High school graduate or less	43 (944)
Some college or higher	57 (922)
**English proficiency (spoken)**	
Poorly or not at all	39 (479)
So-so	40 (812)
Fluently or well	21 (583)
**Years in the United States**	
<10 years	23 (276)
≥10 years	77 (1,491)
**Children in the home**	
No children	39 (873)
≥1 children	61 (1,012)
**Smoking history**	
Never smoker	86 (1,500)
Former smoker	15 (387)
**Secondhand smoke knowledge**	
Not knowledgeable	20 (347)
Knowledgeable	80 (1,529)
**Secondhand smoke attitudes**	
Disapprove or are unsure of ban	11 (170)
Favor smoking ban in bars	90 (1,714)
**Secondhand smoke exposure^b^**	
Home	30 (466)
Work^c^	8 (48)
Bars	52 (140)
College	67 (111)

a Percentages are weighted. Numbers are unweighted. Some strata do not sum to 1,887 because of missing data. Some percentages may exceed 100 because of rounding.

b Computed based on individuals who reported exposure in the setting of interest. For example, the percentage of respondents reporting secondhand smoke exposure at work was computed among participants who were currently employed.

c Because of limited secondhand smoke exposure among women, only men were included in the workplace secondhand smoke variable.

Vietnamese women were more likely than men to be exposed at home (odds ratio [OR], 1.85; 95% confidence interval [CI], 1.24–2.75), but less likely than men to be exposed to SHS at bars (OR, 0.42; 95% CI, 0.18–0.95) (Table 2). Adults aged 40 or older were less likely than those under 40 to be exposed to SHS at home (OR, 0.69; 95% CI, 0.50–0.96). Male former smokers were more likely than male never smokers to have SHS exposure at work (OR, 3.04; 95% CI, 1.27–7.25). No significant correlate was observed for SHS exposure at college.

**Table 2:** Odds of Secondhand Smoke Exposure Among Vietnamese Adults Nonsmokers, California Vietnamese Adult Tobacco Use Survey, 2007–2008

Characteristic	Odds Ratio (95% Confidence Interval)			
	Home (n = 1,575)	Work^a^ (n = 420)	Bars (n = 229)	College (n = 145)
**Sex**				
Male	1 [Reference]			
Female	1.85 (1.24–2.75)^b^	NA	0.42 (0.18–0.95)^c^	1.10 (0.45–2.71)
**Age, y**				
<40	1 [Reference]			
≥40	0.69 (0.50–0.96)^c^	1.82 (0.71–4.68)	0.82 (0.42–1.60)	0.88 (0.35–2.18)
**Education**				
High school graduate or less	1 [Reference]			
Some college or higher	1.02 (0.69–1.49)	0.59 (0.27–1.30)	1.29 (0.44–3.83)	0.65 (0.23–1.82)
**English proficiency (spoken)**				
Poorly or not at all	1 [Reference]			
So-so	0.92 (0.63–1.33)	1.70 (0.53–5.47)	0.71 (0.22–2.34)	1.49 (0.23–9.49)
Fluently or well	1.06 (0.62–1.80)	2.15 (0.62–7.44)	0.40 (0.10–1.64)	2.04 (0.31–13.4)
**Years in the United States**				
<10 years	1 [Reference]			
≥10 years or more	0.74 (0.49–1.13)	0.42 (0.14–1.23)	0.58 (0.21–1.62)	1.13 (0.46–2.79)
**Children in the home^d^**				
No children	1 [Reference]	NA	NA	NA
≥1 children	0.82 (0.59–1.14)			
**Smoking history**				
Never smoker	1 [Reference]			
Former smoker	1.03 (0.66–1.60)	3.04 (1.27–7.25)^b^	1.56 (0.57–4.27)	0.84 (0.15–4.74)
**Secondhand smoke knowledge**				
Not knowledgeable	1 [Reference]			
Knowledgeable	0.84 (0.57–1.23)	0.71 (0.29–1.75)	1.14 (0.49–2.64)	1.00 (0.35–2.88)
**Secondhand smoke attitudes^d^**				
Disapprove or are unsure of ban	NA	NA	1 [Reference]	NA
Favor smoking ban in bars			0.80 (0.18–3.49)	

Abbreviation: NA, not applicable.

a Regression model includes men only.

b *P* < .01.

c *P* < .05.

d Children in the home and secondhand smoke attitudes are included only in regression models for the home and bars, respectively.

## Discussion

Nonsmoking Vietnamese adults in California have high SHS exposure in various settings, despite advanced tobacco control efforts statewide (2). Nearly 1 in 3 adults were exposed in the home. Consistent with a survey in Vietnam (8), women and younger adults are more likely to be exposed at home. Overall, however, SHS exposure at home may be higher in Vietnam than among Vietnamese in California (8), possibly because of California’s history of implementing stringent tobacco control strategies (9) or acculturation stemming from these policies. Eight percent of employed men had SHS exposure at work. Exposure was higher among former than never smokers, which may be due to former smokers selecting workplaces with less strict smoking rules before quitting smoking. More than half of Vietnamese nonsmokers in California who visited a bar in the past 12 months had been exposed to SHS. Exposure was more likely among men, which may be related to higher socializing with male smokers, given social acceptability of smoking among Vietnamese men (3,4). More than two-thirds of Vietnamese nonsmokers taking college classes were exposed to SHS on campus. The study was unable to identify factors associated with exposure at college, suggesting more research is needed.

California’s Comprehensive Tobacco Control Program supports enforcing SHS policies in public places and changing norms through media campaigns, which have been included in CPPW efforts targeting SHS in Vietnamese populations (10). Our findings reveal that Vietnamese nonsmoking adults were knowledgeable about SHS health effects and yet many continue to be exposed to SHS. These findings underscore the need for culturally targeted interventions for Vietnamese adults to reduce SHS exposure. Interventions might include increasing social and cultural acceptance of consistent implementation of smoking bans at home and in other settings, particularly among women, younger adults, and former smokers. Our findings may also have implications for interventions with other Asian populations, given high smoking rates among men and similar cultural norms (11).

Limitations include 1) self-reported data; 2) small numbers of adults exposed in settings other than the home; and 3) unavailability of key determinants of SHS exposure, like occupation. Additionally, data are from 2007–2008. However, a recent survey indicates high smoking rates among Vietnamese men, suggesting that our results have continued relevance (12). Despite these limitations, this is the only in-language population-based survey focused on tobacco-related issues among Vietnamese adults in the United States. Our findings reveal significant SHS exposure among nonsmoking Vietnamese adults in California in various settings, despite advanced tobacco control efforts (2), emphasizing the importance of targeted efforts to prevent SHS exposure for this population.
